# Supplementary material for: Integration of dilated cardiomyopathy genomics with transcriptomics from the human heart implicates regulatory molecular mechanisms
Source: medRxiv. 2026 Jan 13:2026.01.12.26343934. Preprint. [Version 1] doi: 10.64898/2026.01.12.26343934 (PMC12870565; doi:10.64898/2026.01.12.26343934)
Supplement: Supplement 3 [file NIHPP2026.01.12.26343934v1-supplement-3.pdf]

medRxiv preprint doi: https://doi.org/10.64898/2026.01.12.26343934; this version posted January 13, 2026. The copyright holder for this preprint (which was not certified by peer review) is the author/funder, who has granted medRxiv a license to display the preprint in perpetuity.
It is made available under a CC-BY 4.0 International license .

Supplemental Figures

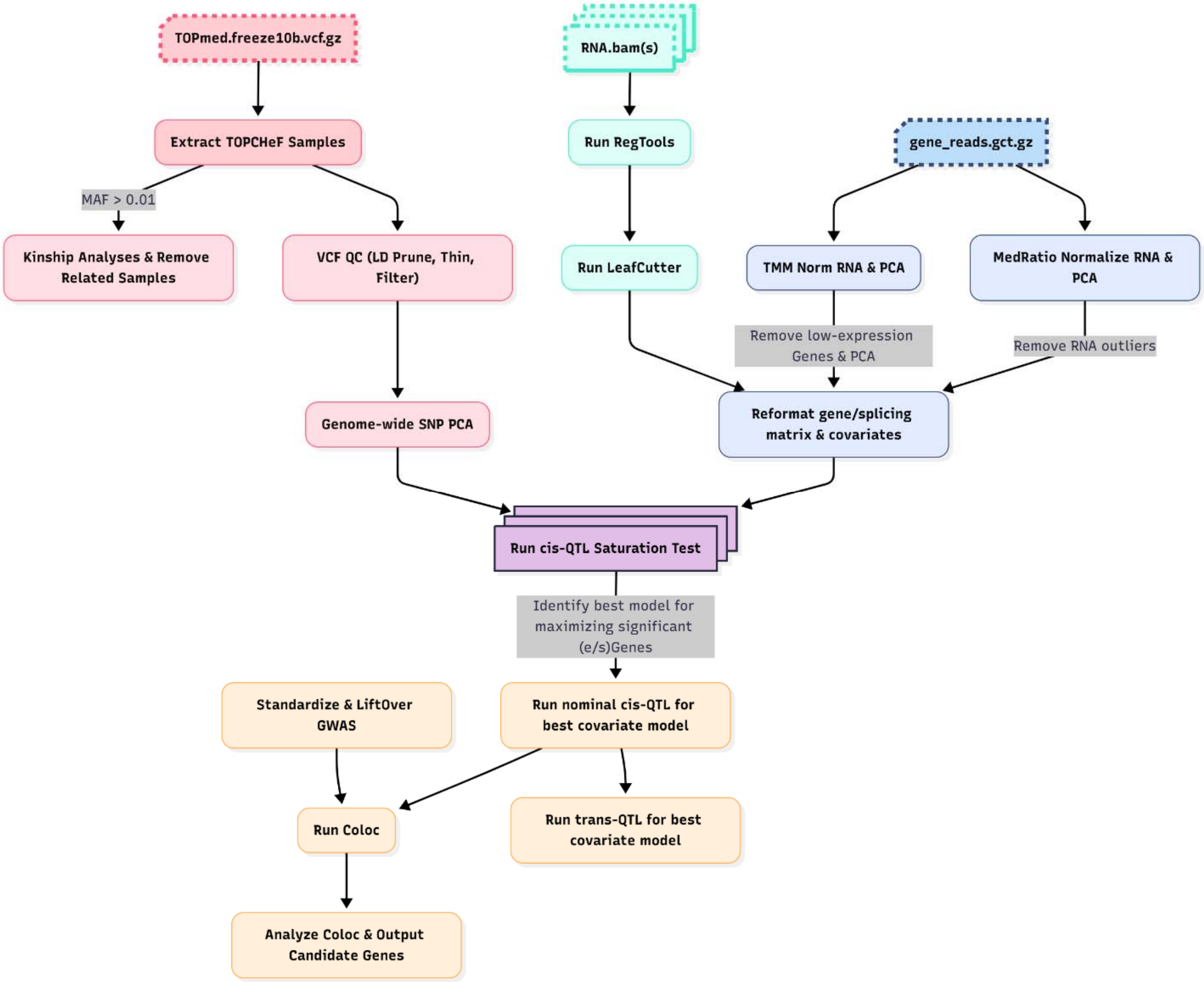

Supplemental Figure 1: ***Computational pipeline for the genetic, transcriptomic, and splicing processing and QTL mapping.*** This pipeline is split between the genetic processing of whole-genome sequencing (pink), RNA-sequencing (blue), and splicing (cyan).

medRxiv preprint doi: https://doi.org/10.64898/2026.01.12.26343934; this version posted January 13, 2026. The copyright holder for this preprint
(which was not certified by peer review) is the author/funder, who has granted medRxiv a license to display the preprint in perpetuity.
It is made available under a CC-BY 4.0 International license .

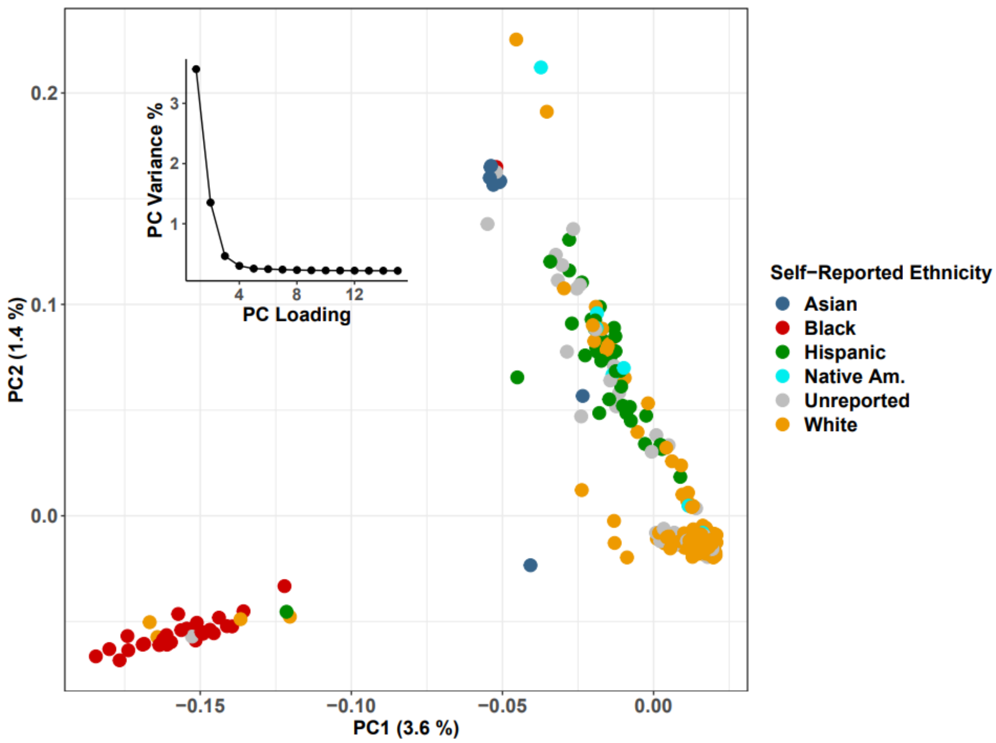

<u>Supplemental Figure 2:</u> ***Genetic variation within TOPCHeF*.** PCA of minor allele frequency filtered (MAF > 0.05) and thinned dataset colored by self-reported ancestry. Inset plot is the proportion of variance explained by each PC loading for the first 15 PCs.

medRxiv preprint doi: https://doi.org/10.64898/2026.01.12.26343934; this version posted January 13, 2026. The copyright holder for this preprint (which was not certified by peer review) is the author/funder, who has granted medRxiv a license to display the preprint in perpetuity.
It is made available under a CC-BY 4.0 International license .

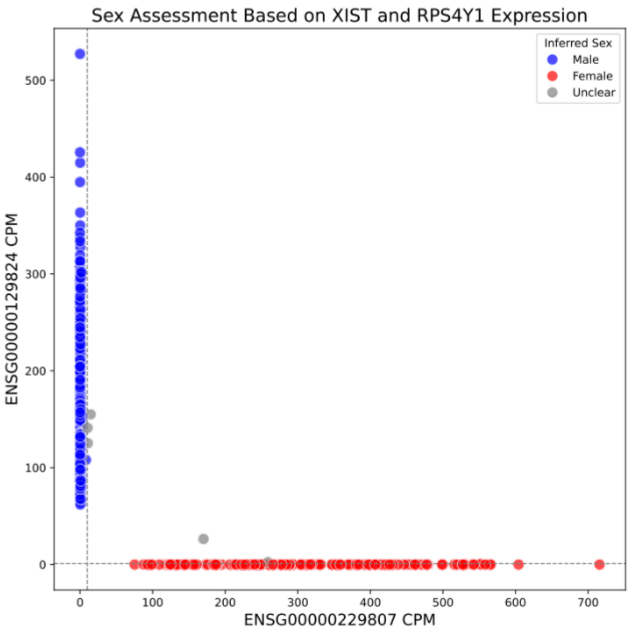

<u>Supplemental Figure 3:</u> ***Assigned sex metadata overlaps with sex determination based on XIST and RPS4Y1 expression.*** Each point is a sample, post-TMM normalization. The three unclear samples were retained because they did not represent clear cases of contamination. ENSG00000229807 (x-axis) is *XIST* and ENSG00000129824 (y-axis) is *RPS4Y1*.

medRxiv preprint doi: https://doi.org/10.64898/2026.01.12.26343934; this version posted January 13, 2026. The copyright holder for this preprint (which was not certified by peer review) is the author/funder, who has granted medRxiv a license to display the preprint in perpetuity.
It is made available under a CC-BY 4.0 International license .

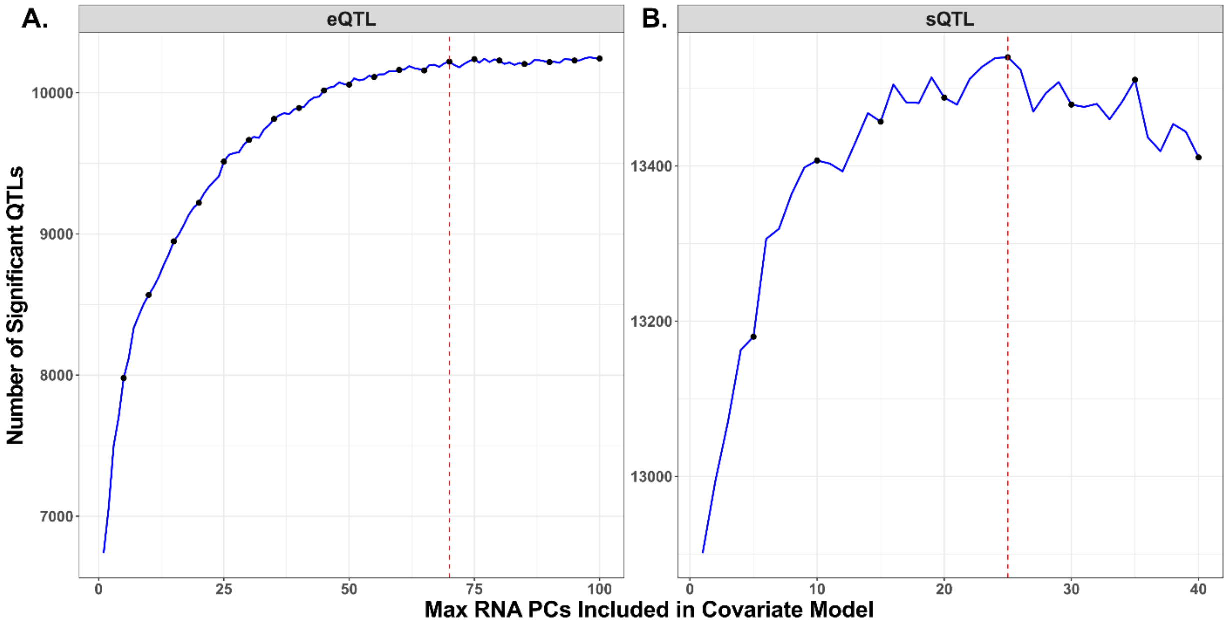

<u>Supplemental Figure 4:</u> ***Saturation of cis-QTL are maximized when including several RNA PCs in the covariate models.*** **A)** Significant eQTL are maximized (i.e., reached a statistical plateau) at RNA PC 70 (red dotted vertical line), we varied the number of maximum PCs included in the covariate model for cis-eQTL. **B)** Significant sQTL are maximized at the RNA PC 25 (red dotted vertical line).

medRxiv preprint doi: https://doi.org/10.64898/2026.01.12.26343934; this version posted January 13, 2026. The copyright holder for this preprint (which was not certified by peer review) is the author/funder, who has granted medRxiv a license to display the preprint in perpetuity.
It is made available under a CC-BY 4.0 International license .

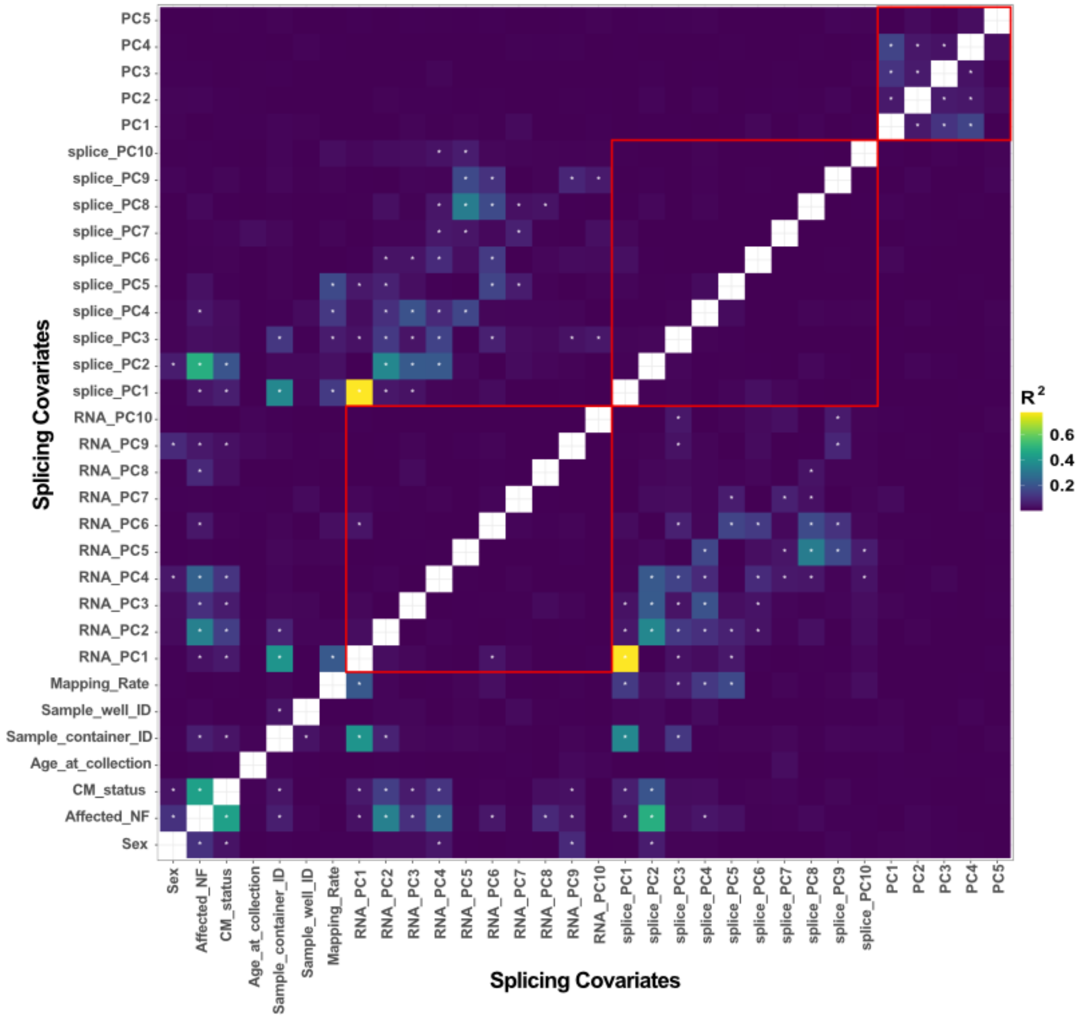

<u>Supplemental Figure 5:</u> ***Pairwise correlation of covariates included in eQTL and sQTL mapping.*** We used the values for every individual and ran Spearman's correlation statistics to estimate a rank-based measure of association. White stars indicate a significant correlation after multiple testing correction (FDR $p < 0.05$). The red box within the matrix indicates any pairwise expression RNA PCs, splicing RNA PCs, or PCs of ancestry comparisons. We added three covariates ("*Sample_container_ID*", "*Sample_well_ID*", and "*Mapping_Rate*") that are absent from the covariate model for QTL mapping but likely reflect experimental batch effects introduced during sample preparation or RNA extraction.

medRxiv preprint doi: https://doi.org/10.64898/2026.01.12.26343934; this version posted January 13, 2026. The copyright holder for this preprint (which was not certified by peer review) is the author/funder, who has granted medRxiv a license to display the preprint in perpetuity.
It is made available under a CC-BY 4.0 International license .

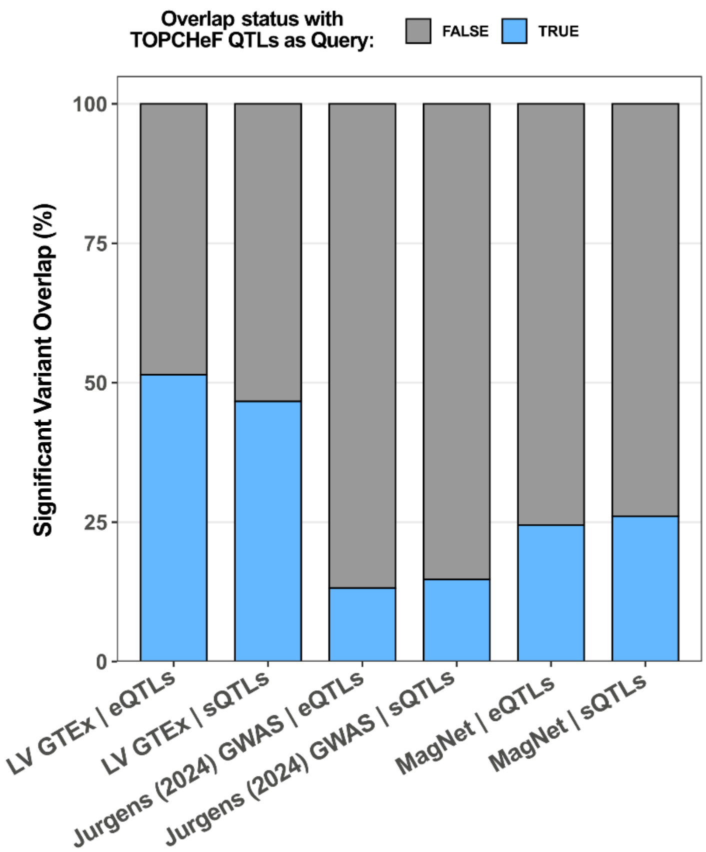

<u>Supplemental Figure 6:</u> ***Overlap replication of TOPCHeF QTL with GTEx, GWAS, and MagNet.*** Proportions of significant eQTL and sQTL that overlap across the three datasets with the significant TOPCHeF QTL.
